# The Lid Wiper Effect on Corneal Epithelial Thickness Pattern in Healthy Eyes as Characterized by Zernike Analysis

**DOI:** 10.1016/j.xops.2026.101133

**Published:** 2026-03-02

**Authors:** Hady Yazbeck, Jad F. Assaf, Jiachi Hong, Yan Li, David Huang

**Affiliations:** Casey Eye Institute, Oregon Health & Science University, Portland

**Keywords:** Blinking, Corneal epithelium, Epithelial remodeling, Lid-wiper effect, Zernike analysis

## Abstract

**Purpose:**

To develop quantitative biomarkers that characterize the effect of upper eyelid motion (lid-wiper effect) during blinking on corneal epithelial thickness.

**Design:**

A retrospective study analyzing corneal epithelium thickness maps through Zernike polynomial decomposition.

**Subjects:**

Three hundred thirty-six maps from 135 healthy eyes of 69 subjects.

**Methods:**

A total of 5-mm diameter epithelial thickness maps were acquired using spectral-domain OCT. Maps from left eyes were mirror-imaged and pooled with right eyes for analysis. Zernike polynomial decomposition was performed, and average coefficients were obtained. The lowest-order Zernike terms with single-angle dependence—tilt and primary coma—were analyzed as vectors to determine the lid-wiper axes. The lid-wiper gradient (μm/mm) and lid-wiper coma (μm) were calculated by projecting the tilt and coma vectors along their lid-wiper axes. Correlation analysis was performed between the lid-wiper gradient and lid-wiper coma, primary astigmatism, and higher-order aberrations.

**Main Outcome Measures:**

The lid-wiper gradient and lid-wiper coma, which are quantitative biomarkers of epithelial remodeling in response to the lid-wiper effect.

**Results:**

The average epithelial thickness map showed superotemporal thinning with relative inferonasal thickening. Average tilt and coma coefficients were nonzero (*P* = 0.004 to <0.001). The population centroids (mean ± standard deviation) of the lid-wiper gradient and lid-wiper coma were 0.51 ± 0.57 μm/mm at 298.06 ± 56˚, and 0.21 ± 0.55 μm at 311.59 ± 83.53˚, respectively. The lid-wiper gradient and coma were positively correlated with each other (*R*^*2*^ = 0.08, *P* = 0.001).

**Conclusions:**

A significant epithelial thickness gradient exists in the average normal cornea, consistent with eyelid blink dynamics described in the literature. The significant but weak correlation between the lid-wiper gradient and coma suggests the lid-wiper effect may be among various factors contributing to higher-order aberrations in the epithelium. The lid-wiper gradient and coma may serve as quantitative biomarkers of epithelial remodeling in response to the lid-wiper effect.

**Financial Disclosure(s):**

Proprietary or commercial disclosure may be found in the Footnotes and Disclosures at the end of this article.

The corneal epithelium is the thin, outermost layer of the cornea that serves as a protective barrier against the outer environment and maintains a smooth anterior corneal surface, enhancing visual acuity and quality.^1^ It also plays a significant role on the anterior corneal surface power.^2^ It has been well established that the corneal epithelium is a dynamic layer, undergoing significant remodeling to minimize irregularity in the anterior corneal curvature, whether from ectatic disorders,^3^ cross-linking,^4^ cataract surgery,^5^ or refractive surgery.^6^ As such, many studies have underscored the importance of understanding and predicting epithelial remodeling behavior. In refractive surgery, for example, this has been used to guide preoperative planning of ablation depth for treatment optimization and minimizing correction regression, as well as postoperative monitoring and guiding retreatment.^7^, ^8^, ^9^ Patterns of epithelial remodeling have also shown great value in diagnosis, screening, and monitoring of corneal pathologies that involve the epithelium, such as keratoconus^3^^,^^10^^,^^11^ and dry eye disease.^12^, ^13^, ^14^

However, there is relatively less literature on how physiologic factors affect the distribution of the corneal epithelium in normal, healthy eyes.^15^ One such factor is spontaneous eye blinking, during which corneal epithelial cells are subjected to mechanical shear stress resulting from the upper eyelid traverse.^15^, ^16^, ^17^ Some studies have explored the dynamics of eyelid motion during blinking.^17^^,^^18^ Others have studied the epithelium distribution in normal eyes and found an overall pattern of supertemporal thinning,^19^^,^^20^ postulating that it could be caused by chafing of epithelial cells from eyelid traverse.^19^ There is also evidence that increased shear stress leads to increased cell shedding on rabbit eyes in vitro.^21^ However, to our knowledge, no research has been done to quantify the direction and magnitude of epithelial thinning in the cornea of healthy eyes in relation to the upper eyelid motion during blinking.

In this study, we leverage the high resolution of OCT^22^ and use Zernike analysis to model the physiologic epithelial distribution in the healthy cornea. We then analyze relevant Zernike terms to describe the magnitude and angular direction of epithelial thickness variation and relate it to the upper eyelid motion described in the literature. This analytical approach potentially provides us with quantitative biomarkers for corneal epithelial thickness variation in relation to upper eyelid action during spontaneous blinking.

## Methods

This retrospective study was performed at the Casey Eye Institute, Oregon Health & Science University and was approved by the Oregon Health & Science University Institutional Review Board. The institutional review board approved the reanalysis of the existing data for algorithm development and waived the requirement for reconsent. Patient data were anonymized to ensure confidentiality. All procedures were in compliance with the Health Insurance Portability and Accountability Act of 1996 and followed the principles of the Declaration of Helsinki.

### Patient Selection

Normal subjects were previously enrolled in a normative imaging database and included volunteers as well as patients seeking refractive or cataract surgery consultation. The present retrospective analysis included all eligible examinations from this database with normal slitlamp microscopy and corneal topography. Eyes with any signs or history of corneal disease, or any prior refractive or other ocular surgery, were excluded.

### OCT Scanning

Corneal maps were acquired using the Visionix (formerly Optovue) Avanti and Solix spectral-domain OCT systems. The Avanti system operates at a wavelength of 840 nm with a scan speed of 70 kHz. It uses a radial scan pattern of 8 sequential and equally spaced meridians, each with 1020 axial scans extending over a 6 mm length. This 8-meridian pattern is repeated 5 times during a single scan with a total scan time of approximately 0.6 s. The Solix system operates at a wavelength of 840 nm with a scan speed of 120 kHz. It uses a radial scan pattern of 16 sequential and equally spaced meridians, each with a length of 10 mm. Both systems have an axial resolution of 5 μm in tissue.

### Data Processing

Epithelium maps of left eyes were mirror-imaged and pooled with those from right eyes for analysis. Multiple scans performed on the same day were averaged to obtain 1 map per eye. Corneal epithelial thickness maps were cropped to the central 5-mm diameter optical zone. Zernike polynomial decomposition was performed up to the fifth radial order and angular frequency using the Prysm library (version 0.21.1) in Python.^23^ Zernike coefficients are reported according to the American National Standards Institute convention. Scans from subjects with outlier values for the Zernike terms of interest were manually reviewed for segmentation errors, and those with identifiable segmentation artifacts were excluded from the analysis. The root mean square error between the epithelium maps and the Zernike-reconstructed maps was used to assess the goodness of Zernike fitting.

### Avanti and Solix Map Compatibility

To confirm the compatibility of Avanti and Solix maps for pooled analysis, Zernike coefficients of maps obtained from 60 eyes of 31 subjects imaged with both devices on the same day were compared. The normality of each coefficient's distribution was assessed using the Shapiro–Wilk test. Normally distributed coefficients were compared using the paired *t*-test, and non-normally distributed coefficients were compared using the Wilcoxon signed-rank test. No statistically significant differences were observed, so the maps from both devices were pooled for analysis.

### Statistical Analysis

Because the upper lid action on the corneal surface is expected to be one of first order asymmetry, the lowest order Zernike coefficients with single-angular dependence—tilt and primary coma—were of interest in this study. For the rest of this manuscript, the X and Y components of the tilt coefficient will be referred to as X-tilt and Y-tilt, respectively, and the X and Y components of primary coma will be referred to as X-coma and Y-coma, respectively. These coefficients were analyzed as vectors and averaged over the study population of healthy eyes. The normality of their distribution was assessed using the Shapiro–Wilk test, and *P* values were calculated to test whether each coefficient was significantly different from zero. Y-tilt and Y-coma followed a normal distribution, whereas the X-tilt and X-coma did not. Therefore, the one-sample *t*-test was used for Y-tilt and Y-coma, while the Wilcoxon signed-rank test was used for X-tilt and X-coma. Bonferroni adjustment was performed for multiple hypothesis testing. Statistical analysis and plotting were performed using the NumPy, SciPy, and Matplotlib Python library packages.^24^, ^25^, ^26^

### Lid Wiper Axis Calculation

The population means for the Y-tilt, X-tilt, Y-coma, and X-coma coefficients (in μm) were obtained. The tilt lid wiper axis (LWA) was derived from the mean tilt vectors using the two-argument arctangent function, atan2 (mean Y-tilt, mean X-tilt). The coma LWA was derived from the mean coma vectors using atan2 (– mean Y-coma, – mean X-coma). The negative sign was used to align the coma vectors with the tilt convention. The circular standard deviation (SD) for those axes was also computed.^27^

### Lid Wiper Gradient Calculation

For each eye, the lid wiper gradient (in μm/mm) was calculated by projecting its tilt vector onto the tilt LWA, using the following formula:

$$Lid\;Wiper\;Gradient=\frac{\text{tilt}\;\text{magnitude}\cdot \cos\left(\Delta \theta_{\text{tilt}}\right)}{r}$$

Where r is the radius of the analytical zone (2.5 mm), and $\Delta \theta_{\text{tilt}}$ is the angular difference between the eye's tilt vector axis and the tilt LWA. The average total vector (centroid) magnitude and orientation of the lid wiper gradient was then calculated along with its 95% confidence ellipse.

### Lid Wiper Coma Calculation

For each eye, the lid wiper coma (in μm) was calculated by projecting its coma vector onto the coma LWA, using the following formula:

$$Lidwiper\;coma=\text{coma}\;\text{magnitude}\cdot \cos\left(\Delta \theta_{\text{coma}}\right)$$

Where $\Delta \theta_{\text{coma}}$ represents the angular difference between the eye's coma axis and the coma LWA. Note again that a negative sign was applied to the coma vectors to flip their direction and match the tilt convention. The average total vector (centroid) magnitude and orientation of the lid wiper coma was then calculated along with its 95% confidence ellipse.

### Correlation Analysis

Linear regression was performed to assess the correlation between the lid wiper gradient and lid wiper coma, root-sum-square of primary astigmatism, and root-sum-square of higher-order aberrations (excluding primary coma).

## Results

**Figure 1:**
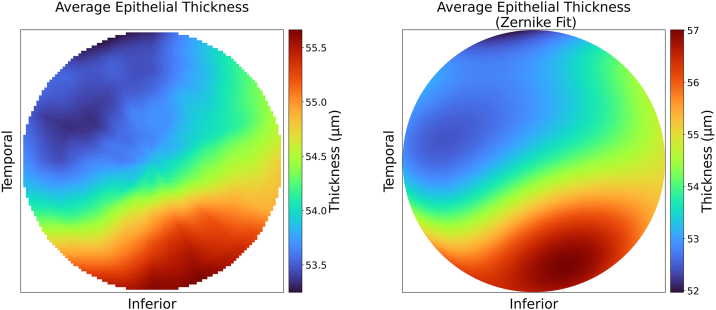
Population-averaged 5-mm corneal epithelial thickness map and corresponding Zernike-reconstructed map. Left: Population average of the 5 mm diameter corneal epithelium thickness map. Right: 5-bb mm diameter corneal epithelium thickness map reconstructed from population averages of Zernike coefficients, up to the fifth radial and azimuthal order polynomials. Thickness measurements are in μm.

Our study included a total of 336 OCT epithelium maps from 135 healthy eyes of 69 patients: 35 females with mean age ± SD of 37.3 ± 10.9 years, and 34 males with mean age ± SD of 37.5 ± 9.5 years. The mean ± SD of the root mean square error between the epithelium maps and the Zernike-reconstructed maps was 0.35 ± 0.11 μm. The mean Zernike fit compared to the population average of all epithelium maps is represented in Figure 1.

**Figure 2:**
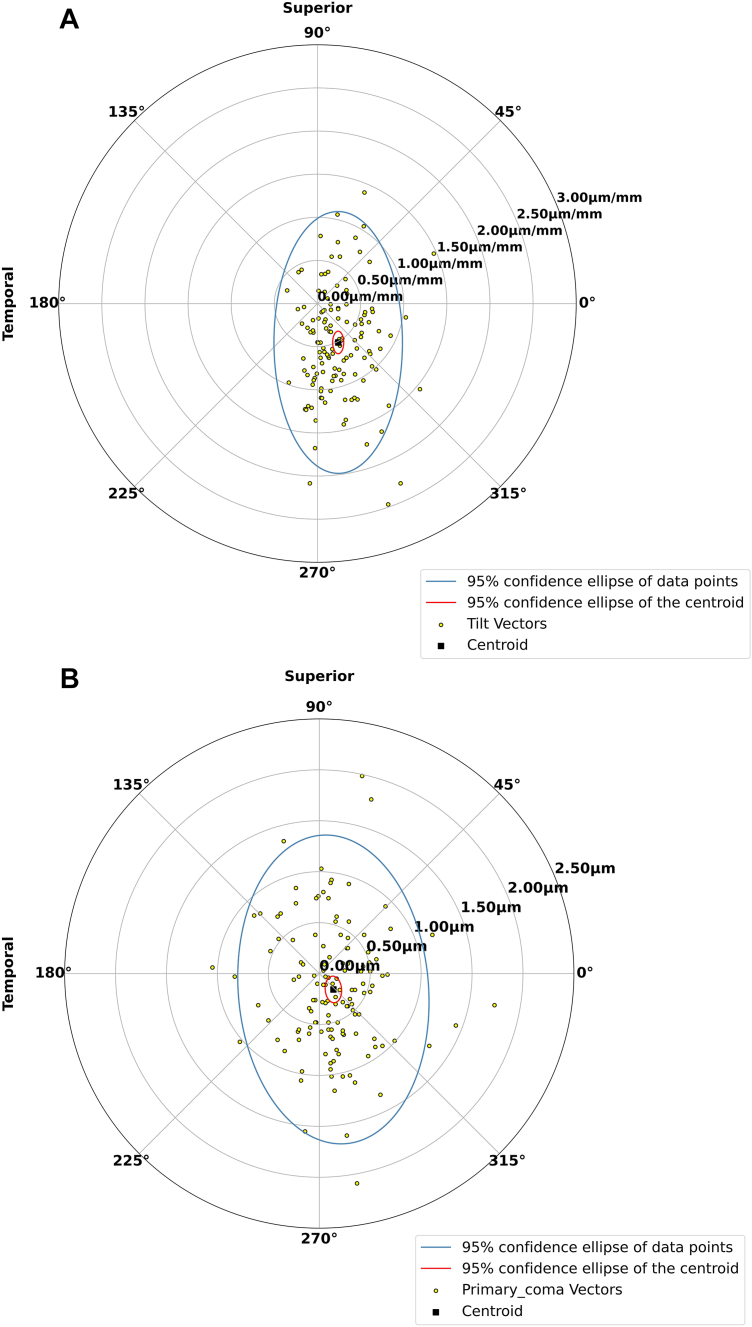
Polar plots showing the magnitudes and axes of the lid wiper gradient **(A)** and lid wiper coma **(B)**. Yellow dots are the data points (each representing 1 eye), the black square is the centroid of the data points, the red line is the 95% confidence ellipse of the centroid, and the blue line is the 95% confidence ellipse of the data points.

The population mean and SD of the tilt and coma coefficients are shown in Table 1. The mean ± SD coefficients of the Y-tilt and X-tilt terms were –1.13 ± 1.57 μm and 0.60 ± 0.80 μm, respectively. The centroid of the lid wiper gradient was 0.51 ± 0.57 μm/mm at 298.06˚ ± 56˚ (Fig 2A). This represents superotemporal (118.06˚) epithelial thinning with relative inferonasal (298.06˚) thickening at an average gradient of 0.51 μm/mm.

**Table 1:** Epithelial Zernike Terms with Single Angular Frequency

Zernike Term	Mean (μm)	Standard Deviation (μm)	*P* Value
Z(1, –1), Y-tilt	–1.13	1.55	<0.001
Z(1, 1), X-tilt	0.60	0.76	<0.001
Z(3, –1), Y-coma	0.16	0.62	0.004
Z(3, 1), X-coma	–0.14	0.38	<0.001

The mean ± SD coefficients of the Y and X coma vectors were 0.16 ± 0.61 μm and –0.14 ± 0.38 μm, respectively. The centroid of the lid wiper coma was 0.21 ± 0.55 μm at 311.59 ± 83.53˚ (Fig 2B). This represents a pattern of paracentral focal superotemporal (131.59˚) thinning with relative inferonasal (311.59˚) thickening.

All results were statistically significantly different from 0 (*P* = 0.004 to <0.001).

As for correlation analysis, lid wiper gradient and lid wiper coma were weakly but significantly correlated with a slope of 0.26 mm, a Pearson correlation coefficient of 0.27, and an *R*^*2*^ value of 0.08 (*P* = 0.001). No statistically significant relationship was detected between the lid wiper gradient and the root-sum-square of primary astigmatism (slope = 0.16 mm, Pearson correlation coefficient = –0.16, *R*^*2*^ = 0.03, *P* = 0.06) or higher-order aberrations excluding primary coma (slope = 0.05 mm, Pearson correlation coefficient = 0.08, *R*^*2*^ = 0.006, *P* = 0.36).

## Discussion

In the present study, we were able to accurately reconstruct epithelium thickness maps using Zernike polynomial decomposition. This approach allows us to analyze patterns of epithelial thickness distribution and quantify physiologic trends in the healthy corneal epithelium. In particular, the tilt terms revealed an average lid wiper gradient of 0.51 μm/mm at an angle of 298.06˚, representing relative epithelial thinning from the inferonasal to superotemporal direction. This lid wiper gradient is consistent with the shear stress and pressure exerted on the anterior ocular surface by the upper eyelid during blinking,^17^^,^^28^, ^29^, ^30^ as well as the upper eyelid motion described in the literature.^17^^,^^18^ Wambier et al^18^ measured the horizontal and vertical components of the upper eyelid blink using a portable video system that tracked the upper eyelid. They found that the horizontal movement was approximately 40% of the vertical component of the blink amplitude. This corresponds to a diagonal blinking motion at an angle of 291.8˚, in agreement with the axis we report for the lid wiper gradient. In conjunction, Reinstein et al^19^ reported superotemporal thinning of the corneal epithelium within a 10-mm diameter in a population of 110 normal eyes of 56 patients, using high-frequency ultrasound. Within a 6-mm diameter, they found that the thinnest epithelium point was located on average 0.33 mm temporally and 0.90 mm superiorly. This translates into an average angle of 110˚ superotemporally. Though they do not compute gradients across the corneal epithelium, their observations suggest a lid wiper gradient at a 290˚ axis, consistent with our findings. Similarly, another study done on 67 healthy North Indian eyes of 67 subjects found that the epithelial thickness, measured using spectral-domain OCT, was significantly thinner in the superior and superotemporal sectors as compared to their radially opposite sectors within a 5-mm diameter.^20^

To our knowledge, our study is the first to quantify the direction and magnitude of the epithelial thickness gradient, resulting from the lid-wiper effect, and relate it to the upper eyelid motion described in the literature. Our findings may offer valuable insights into the impact of blink dynamics on epithelial remodeling. However, the wide variation in the lid wiper axes and magnitudes observed in our data suggests that the lid wiper effect only partially explains the epithelial thickness distribution seen in normal corneal epithelium. Other mechanisms, such as limbal cell health and migration, as well as epithelial smoothing in response to corneal curvature, likely play significant roles in shaping the epithelial thickness distribution.^31^^,^^32^ Several factors at the individual level may also influence the lid wiper effect on epithelial remodeling, increasing variation. These include eyelid geometry, tightness, and inner surface roughness, blink velocity and amplitude, tear film quality and volume, corneal shape, and degree of globe protrusion. Each of these variables can alter the mechanical interaction between the eyelid's inner surface and the corneal epithelial surface. Therefore, the lid wiper gradient may serve as a quantitative biomarker for various ocular conditions, such as eyelid surgery (affecting lid geometry and tension), chronic conjunctival inflammation (increasing eyelid surface irregularity and friction), dry eye disease (disrupting the tear film and increasing friction), corneal refractive surgery (altering corneal shape), and thyroid eye disease (increasing globe protrusion and eye lid pressure).

Another interesting finding is the focal epithelial superotemporal (131.59˚) thinning with relative inferonasal (311.59˚) thickening represented by the lid wiper coma. This shows that the lid wiper gradient is only a rough approximation of lid wiper effect, which can be appreciated on the epithelial thickness pattern in Figure 1 showing a more pronounced gradient in the temporal part of the cornea, compared to nasal. This may be explained by the fact that the blink motion is not exactly linear but slightly arcuate, according to the blink patterns described by Wambier et al.^18^ Doane also reported that the upper eyelid accelerates rapidly and reaches its maximum speed by the time it crosses the visual axis, before decelerating to rest as it draws closer to the lower lid. Consequently, the upper lid blink motion should have larger excursion and velocity in the temporal half of the cornea compared to the nasal half. The nonlinear nature of spontaneous blinking may explain why the lid-wiper effect could induce higher-order aberration in the epithelium, and why the lid-wiper coma axis is more temporally oriented compared to that of the tilt. The significantly positive but weak correlation between the lid wiper gradient and lid wiper coma terms suggests that the lid wiper effect may be one of several factors contributing to higher order aberrations in the epithelium. Further investigation of this relationship is warranted.

We hypothesize that Zernike-derived epithelial biomarkers, including the lid wiper gradient and lid wiper coma, may provide a quantitative framework for evaluating how interventions that modify eyelid–ocular surface mechanics influence epithelial remodeling. For instance, lubrication therapies for dry eye disease may be expected to decrease the magnitude of the lid wiper gradient and coma. Blepharoplasty or botulinum toxin treatment, which can alter eyelid geometry, blink dynamics, and lid tension, may result in measurable changes in these biomarkers. Similarly, refractive procedures such as LASIK and photorefractive keratectomy may modify the eyelid-surface interaction by altering corneal shape, potentially affecting the intensity of the lid-wiper effect postoperatively. Exploring the relationship between these interventions and the lid wiper gradient and coma will be an important objective for future prospective studies.

There are limitations to our study. Firstly, given the ∼3 μm thickness of the tear film and the 5 μm axial resolution of the OCT system used, the tear film could not be separately resolved in our measurements.^33^ However, prior literature suggests that tear film thickness is unlikely to confound our results. Current evidence on *in vivo* tear film thickness distribution supports a predominant pattern of superior thickening relative to inferior.^34^^,^^35^ Moreover, Reinstein et al^19^ studied the corneal epithelial thickness pattern using ultrahigh-resolution ultrasound imaging with immersion scanning (i.e., the tear-film was not included in the epithelial thickness measurement). The average epithelial thickness profile they reported is similar to ours. Another limitation is that our findings are based on single-center data set, which may limit generalizability. Nevertheless, the epithelial thickness distribution we observed is strongly supported by prior evidence in literature, lending confidence to our conclusions.

## Conclusion

In summary, by applying Zernike decomposition to OCT epithelial thickness maps, we quantified the magnitude and direction of a significant epithelial thickness gradient within the average normal cornea. The lid wiper gradient, describing a relative superotemporal (118.06˚) thinning with inferonasal (298.06˚) thickening, is consistent with the biomechanics of spontaneous blinking described in the literature. Additionally, the significant lid wiper coma with weakly positive correlation with the lid wiper gradient suggests that the lid wiper effect may be linked to higher-order aberrations of the corneal epithelium. Therefore, Zernike analysis of epithelial thickness may provide us with quantitative biomarkers to better understand and describe epithelial remodeling. These biomarkers can potentially be incorporated into broader models describing epithelial remodeling behavior and may be valuable in monitoring conditions affecting the eyelid–epithelium interaction.
